# Optimization of a rat lumbar IVD degeneration model for low back pain

**DOI:** 10.1002/jsp2.1092

**Published:** 2020-06-22

**Authors:** Juliane D. Glaeser, Wafa Tawackoli, Derek G. Ju, Jae H. Yang, Linda EA Kanim, Khosrowdad Salehi, Victoria Yu, Evan Saidara, Jean‐Phillipe Vit, Zhanna Khnkoyan, Zachary NaPier, Laura S. Stone, Hyun W. Bae, Dmitriy Sheyn

**Affiliations:** ^1^ Orthopaedic Stem Cell Research Laboratory Cedars‐Sinai Medical Center Los Angeles California USA; ^2^ Board of Governors Regenerative Medicine Institute Cedars‐Sinai Medical Center Los Angeles California USA; ^3^ Department of Orthopedics Cedars‐Sinai Medical Center Los Angeles California USA; ^4^ Department of Surgery Cedars‐Sinai Medical Center Los Angeles California USA; ^5^ Biomedical Imaging Research Institute Cedars‐Sinai Medical Center Los Angeles California USA; ^6^ Department of Biomedical Sciences Cedars‐Sinai Medical Center Los Angeles California USA; ^7^ Department of Orthopedic Surgery Korea University Guro Hospital Seoul South Korea; ^8^ McGill University, Faculty of Dentistry Alan Edwards Centre for Research on Pain Montreal Canada

**Keywords:** degeneration, inflammation, pain, preclinical models

## Abstract

**Introduction:**

Intervertebral disc (IVD) degeneration is often associated with low back pain and radiating leg pain. The purpose of this study is to develop a reproducible and standardized preclinical model of painful lumbar IVD degeneration by evaluation of structural and behavioral changes in response to IVD injury with increasing needle sizes. This model can be used to develop new therapies for IVD degeneration.

**Methods:**

Forty‐five female Sprague Dawley rats underwent anterior lumbar disc needle puncture at levels L4‐5 and L5‐6 under fluoroscopic guidance. Animals were randomly assigned to four different experimental groups: needle sizes of 18 Gauge (G), 21G, 23G, and sham control. To monitor the progression of IVD degeneration and pain, the following methods were employed: μMRI, qRT‐PCR, histology, and biobehavioral analysis.

**Results:**

T1‐ and T2‐weighted μMRI analysis showed a correlation between the degree of IVD degeneration and needle diameter, with the most severe degeneration in the 18G group. mRNA expression of markers for IVD degeneration markers were dysregulated in the 18G and 21G groups, while pro‐nociceptive markers were increased in the 18G group only. Hematoxylin and Eosin (H&E) and Alcian Blue/Picrosirius Red staining confirmed the most pronounced IVD degeneration in the 18G group. Randall‐Selitto and von Frey tests showed increased hindpaw sensitivity in the 18G group.

**Conclusion:**

Our findings demonstrate that anterior disc injury with an 18G needle creates severe IVD degeneration and mechanical hypersensitivity, while the 21G needle results in moderate degeneration with no increased pain sensitivity. Therefore, needle sizes should be selected depending on the desired phenotype for the pre‐clinical model.

## Graphical abstract

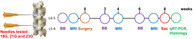

Study Design Overview

This study establishes and validates a rat model for lumbar IVD degeneration that is reflected in painful behavior. Based on our findings we conclude that annular disc injury with a 18G needle creates severe, painful IVD degeneration, while 21G needle injury results in moderate degeneration.

## 1 INTRODUCTION

Low back pain is a leading cause of disability and morbidity in the adult population, affecting ∼80% of adults within their lifetime.^1^, ^2^ Up to 40% of all low back pain is attributed to discogenic pain from intervertebral disc (IVD) degeneration.^3^, ^4^ While the pathophysiology of IVD degeneration is not fully understood, changes in the biological and morphological features as well as biomechanical function are known to be involved in this progressive disease.^5^, ^6^

The IVD consists of a central nucleus pulposus (NP), an outer annulus fibrosus (AF), and two vertebral endplates that connect the soft tissue with the vertebral body.^7^ The NP plays a crucial role in the initiation and progression of IVD degeneration. Various studies demonstrated an increase in inflammatory and catabolic factors and a reduction in anabolic factors in degenerated IVDs in humans,^8^, ^9^ and animal models.^6^, ^10^ For example, an upregulation of matrix metalloprotease‐(MMP)‐3 and a reduction of Collagen II in NP tissue obtained from degenerated discs has been shown.^11^, ^12^ Moreover, reduction of NP hydration as a result of IVD degeneration can be shown using quantification of T2‐weighted images obtained via magnetic resonance imaging (MRI).^13^

Degenerative changes in the IVD are often associated with nerve ingrowth and hyper‐innervation, whereas in normal IVDs only the periphery of the AF is innervated by nerve fibers.^14^ In painful IVD degeneration, higher concentrations of nerve growth and innervation‐related peptides compared to painless degenerative IVDs were shown.^15^, ^16^ For example, IVDs obtained from patients with severe back pain and reduced disc height showed an increased amount of nerve fibers in the endplate region, which were immunoreactive for neuropeptides, such as Substance P.^15^ This suggests that discogenic pain may be a result of changes in innervation. These changes have been shown to be induced by pro‐inflammatory cytokines, including TNF‐α.^17^, ^18^ The importance of TNF‐α in the induction of painful IVD degeneration has been recently shown in a study demonstrating that inhibition of TNF‐α at the time of induced IVD injury limits long‐term pain and degeneration in a rat model.^18^ While imaging‐based diagnosis including MRI provides detailed images of the IVD tissue, it fails to clearly differentiate between a pathologically painful disc and a disc that does not generate pain.^19^, ^20^ Due to the shortcomings of these imaging techniques, IVD degeneration models need to be additionally characterized by assessment of pro‐nociceptive markers and pain‐related behavior.

To date, there are no clinically validated methods for preventing or reversing IVD degeneration and associated discogenic pain. Current nonsurgical treatments include physical therapy and spinal injections with steroids for pain reduction.^21^ Surgical treatments include spinal fusion or arthroplasty procedures to improve stabilization and to reduce pain.^22^ Recently, injections of stem cells, growth factors and anti‐inflammatory factors are being investigated as potential therapies to attenuate the progression of IVD degeneration.^18^, ^23^, ^24^ Prior to testing these treatment modalities in the clinic, small rodent and large animal preclinical models are essential for proof‐of‐concept testing.^25^

Mouse models provide the advantage of using transgenic animals as a powerful tool for mechanistic questions that can isolate parameters like single gene knockout^26^, ^27^, ^28^ or aging.^29^, ^30^ Large animal models provide the size relevant to human tissues for more translational studies.^31^, ^32^, ^33^, ^34^ The rat model, due to its relatively small size, be used in large cohort studies, has relatively large discs compared with the mouse model that allows for injection of therapeutic agents, and supports biobehavioral testing which is a crucial component in studying discogenic pain.^18^, ^25^

To assess the progression of IVD degeneration and to test new treatments in rat models, disc needle puncture is frequently used to induce IVD degeneration, and has been previously performed in tail and lumbar discs.^35^, ^36^ The tail IVD puncture has the advantage that no spinal surgery is needed and that the needle can be inserted into the tail discs percutaneously. However, it does not take into account the anatomical and mechanical conditions of lumbar spine, the main site of IVD degeneration.^37^ On the other hand, injuring the rat lumbar IVD requires an open surgical approach and can be more variable due to harder access. To ensure accurate and reproducible needle insertion into the targeted disc during lumbar spine surgery, use of fluoroscopy has been shown to be a valuable tool to capture of interoperative radiographic images in real time.^38^ In addition to the differences in the spinal locations chosen for needle injury, several other factors largely vary between studies, including the needle diameter used, needle insertion site, path of insertion (eg, ventral vs lateral), depth of needle insertion and number of punctures per disc.^39^ Controlling multiple factors contributing to the degeneration process is crucial to achieve consistent results and to allow for comparison between different studies.

There is multiple evidence of the efficacy of needle injury in inducing IVD degeneration and discogenic pain in a rat model, as shown by MRI, gene expression analysis, histology, and biobehavioral testing.^18^, ^36^, ^40^, ^41^ However, to our knowledge no comprehensive qualitative and quantitative assessment of the changes in the rat lumbar IVD structure, function and pain behavior in response to injury with different needle diameters has been published to date.

In this study, we hypothesize that there is a correlation between needle diameter used for rat lumbar IVD injury, IVD degeneration, and pain‐related behavioral measures. Using a highly controlled surgical approach, we aim to demonstrate how lumbar IVD injury with increasing needle diameters affects disc dehydration, catabolic and anabolic factor regulation, disc morphology, as well as structural and functional pain in a rat model. The results of this study will not only help to create a reliable model to mimic moderate and severe disc degeneration but will also highlight differences across outcome measures related to the severity of IVD degeneration. As an additive value, the needle size that will not induce noticeable degeneration, may be marked as the maximal size that can be used for injection therapy to the disc.

## 2 MATERIALS AND METHODS

### 2.1 Study design

**FIGURE 1:**
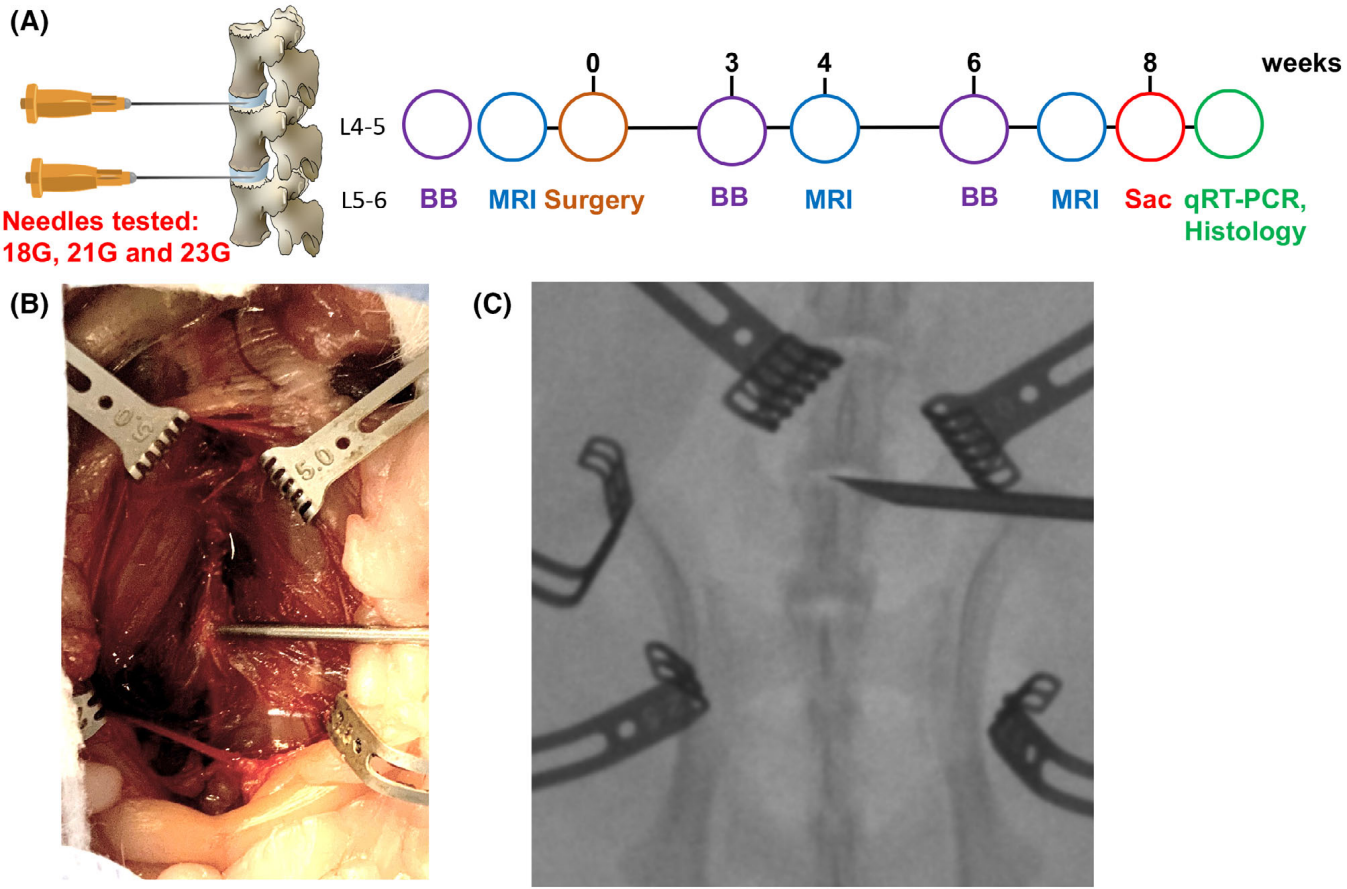
Study design and surgical approach. A, In an anterior surgical approach, lumbar discs L4‐5 and L5‐6 were injured with different needle sizes (18G, 21G, and 23G). Per rat, only one needle size was inserted at each of two levels. Sham operated animals with no IVD injury served as controls. Prior to surgery and at 4‐ and 8‐weeks post‐surgery μMRI images were taken. Biobehavioral tests were performed prior to surgery and at 3‐ and 6‐weeks postsurgery. After sacrifice at 8 weeks, spines were explanted and the spinal segments injured and uninjured L4‐5, L5‐6 were analyzed via qPCR and histology. Sac, day of sacrifice; BB, biobehavioral testing. B, Images of the anterior transperitoneal approach to the lumbar spine. C, A needle is inserted into the exposed lumbar disc L5‐6. Radiographic image of a rat lumbar spine obtained during surgery

Forty‐five healthy, female CD Sprague Dawley IGS rats (Charles River, Massachusetts), 10 to 12 weeks of age underwent anterior lumbar disc surgery in accordance to the institutional IACUC protocol (IACUC008089). Animals were randomly assigned to four different experimental groups: rats injured with needle sizes of 18 gauge (G) (n = 10), 21G (n = 11), 23G (n = 12), and rats that had sham surgery only (n = 12) (Figure 1A). 18G and 21G needle sizes were chosen, since they were used in the past to induce IVD degeneration in tail and lumbar rat IVD without the requirement of additional intradiscal injections.^36^, ^40^, ^42^ We added the 23G needle group to see if a smaller needle size would be sufficient to induce IVD degeneration in the lumbar spine as shown in rat caudal discs.^36^ Under anesthesia and after incision, the anterior lumbar spine was exposed through a transperitoneal anterior approach (Figure 1B). Under fluoroscopic guidance, each needle was inserted into the middle of the nucleus pulposus (NP, total depth of 2 mm, Figure 1C) of lumbar discs L4‐L5 and L5‐L6, held for 5 seconds and removed. Afterwards, the incision was closed, and warm fluids, and pain medication were administered. L4‐5 and L5‐6 were chosen for injury, since we aimed to investigate disc levels that best reflect the situation in humans: L4‐5 and L5‐S1 (L6 does not exist) are the discs that are most often degenerated and painful in humans.^43^ Each of those discs has a unique size, and is exposed to different loading conditions that in turn affect the biological function of each IVD, as seen by a different frequency of degeneration per disc in patients.^43^ To monitor the progression of degeneration of the matching spinal segments in the lumbar spine of the different animals, the following methods were employed: T1‐ and T2‐weighted *μ*MRI analysis performed presurgery and at 4‐ and 8‐weeks postsurgery, biobehavioral analysis done presurgery and at 3‐ and 6‐weeks postsurgery, qPCR to detect IVD degeneration and pain (MMP3 [catabolic marker], aggrecan, collagen II [anabolic makers], collagen I and CCN2 [fibrosis markers], TAC1 and TNFα [pro‐nociceptive markers]), as well as histological stains (H&E and Alcian blue/Picrosirius Red) performed at week 8 postsurgery (Figure 1A).

### 2.2 Animal surgery

Animal experiments were performed in accordance to Cedars‐Sinai's Institutional Animal Care and Use Committee‐approved protocol **(**IACUC008089). Briefly, under inhalation anesthesia and after incision, an anterior transperitoneal approach to the lumbar spine was utilized. An abdominal vertical incision (∼5 cm) was made at the level of the lumbar spine and blunt dissection surgical techniques were used to extend the exposure into the abdominal cavity (Figure 1B,C). The intestines were deflected to the right to expose the abdominal aorta and the left kidney. Anatomical landmarks were then palpated to determine the spinal region to be exposed in upper caudal vertebrae. The anterior edges of the spinal column were isolated from connective tissue and muscle. Prior to puncture, a mini C‐arm was used to clearly identify the level of each IVD (Figure 1C). Using one Gauge (G) of sterile needle per animal (18G, 21G, or 23G), a disc puncture of 2 mm in depth (to the middle of the IVD) was created in two levels L4‐L5 and L5‐L6. The desired insertion depth of 2 mm was ensured by surface marking of the needle tip. After the puncture was completed, homeostasis was assured, the peritoneal contents were replaced, and the peritoneum, rectus fascia, and skin were closed in layers. Sham animals underwent the same surgery, but without IVD needle injury. After surgery, the incision was closed, and warm fluids and pain medication (0.05 mg/kg buprenorphine, SC) were administered. Twelve hours after surgery, application of pain medication (0.05 mg/kg buprenorphine, SC) was repeated. Rats were single housed after surgery to minimize risk of injury through companions. The rats' welfare was assessed daily. No infections, poor conditions, or drug related adverse events were detected.

### 2.3 Micro‐magnetic resonance imaging

**FIGURE 2:**
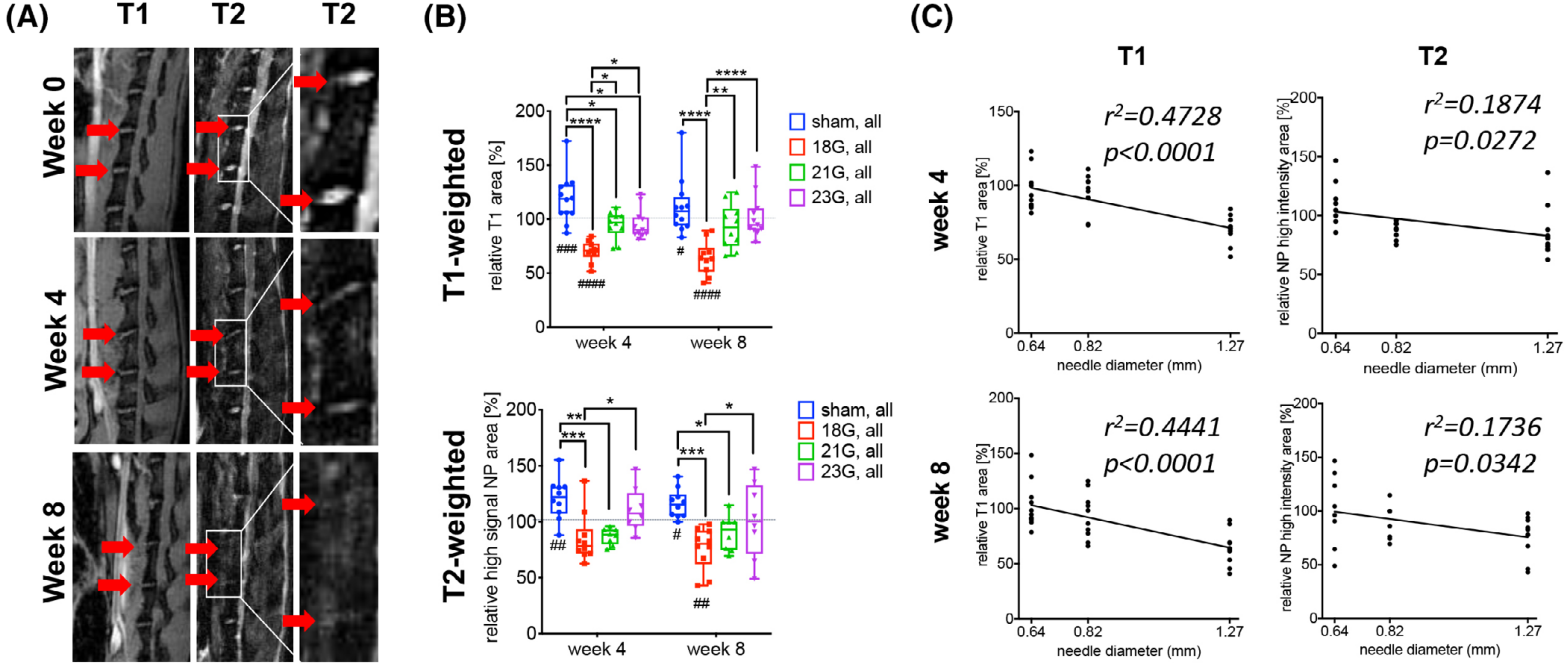
Injury with a 18G needle results in the most severe disc degeneration: μMRI analysis. A, Representative images of 18G needle injured IVDs L4‐5 and L5‐6 over time. IVD degeneration is evidenced by the reduced signal of T1 and T2 area. Left and middle images show lumbar spine segments L2‐S1. The right images show a magnification of L4‐5 and L5‐6. B, Quantitative analysis results of T1‐weighted area and high signal NP area (T2‐weighted) analysis at 4‐ and 8‐ weeks postinjury relative to presurgery. Results are displayed for levels L4‐5 and L5‐6 combined (sham, 18G, 21G, and 23G injured groups). *Indicates statistical differences between different needle sizes. ^#^Indicates statistical differences between different time points. Results are presented as mean ± SD; *(^#^) *P* < .05, **(^##^) *P* < .01, ***(^###^) *P* < .001, ****(^####^) *P* < .0001. C, Regression of disc degeneration parameters (μMRI**)** on needle diameters (mm). Shown is the reduction of T1 area and NP high intensity area (T2) of discs injured with different needle diameters (0.64 mm/23G, 0.82 mm/21G and 1.27 mm/18G) at 4‐ and 8‐weeks postsurgery. Needle diameter (mm) explained 44% to 47% of the variability in the T1 area and 17% to 19% of the variability in NP hydration. μMRI data were normalized to uninjured discs of the same level from rats undergoing sham surgery

To visualize the IVD structure and level of hydration, *μ*MRI imaging, a small animal magnetic resonance imaging scanner, Bruker BioSpec 9.4 T (94/20) with Avance III electronics 9.4 T was used, as previously described by our group.^42^ Micro‐magnetic resonance imaging (Micro‐MRI) was performed at the Imaging Core facility under the Imaging Core's approved IACUC protocol (#007376, Core protocol: μMRI Imaging for Rat Research). Each rat underwent μMRI preoperatively, and at 4 and 8 weeks postoperatively. Time points for MRI imaging and biobehavioral assays were chosen based on prior literature on the evaluation of IVD degeneration in rat models.^18^, ^41^, ^42^ Briefly, anesthetized rats were placed on the examining bed in the prone position. To ensure the optimal angle for sagittal slice scanning, a series of axial, coronal and sagittal pilot proton density (T1) scans (TR: 50 ms, TE: 1.7 ms) were performed. After obtaining satisfactory sagittal midsection proton density scans for outlining the disc location and size, sagittal proton density scans (TR: 50 ms, TE: 1.7 ms) and T2‐weighted scans (TR: 5000 ms, TE: 30 ms) with the exact same imaging geometries were performed. The level of disc hydration was quantitatively measured using MIPAV computer imaging software (Medical Image Processing, Analysis, and Visualization, NIH, Bethesda, Maryland). Utilizing the iliac crest as the anatomical landmark for each scan, regions of interest (ROIs) of IVD L4‐5 and L5‐6 were manually contoured by an independent clinician researcher that was blinded to the conditions for measurements of changes in the disc area (T1‐weighted) and high signal area values of the NP (T2‐weighted). In the figure, relative disc area (T1‐weighted) and high signal area (T2‐weighted) values of injured and uninjured NPs post‐surgery compared with presurgery are shown (Figure 2).

### 2.4 Gene expression analysis

Total RNA was isolated from IVDs using TRIzolReagent (Thermo Fisher Scientific) and subsequent homogenization. RNA extraction was done from whole IVD, including the NP and AF, but excluding endplates. Six independent samples (3 × L4‐L5 and 3 × L5‐L6) were used for RNA extraction and analysis. IVDs were explanted right after rats' euthanasia, shock frozen in liquid nitrogen and stored at −80°C until processing. RNA was isolated via chloroform extraction and RNA cleanup was performed with Qiagen RNeasy mini kit (Qiagen, Germantown Maryland). RNA quality and quantity was determined via spectrophotometry. RNA was transcribed in cDNA using the High‐Capacity cDNA Reverse Transcription Kit (Applied Biosystems). Levels of rat MMP3, aggrecan, collagen1α1 and collagen2α1, CCN2, TAC1, and TNF‐α (Rn00591740_m1, Rn00573424_m1, Rn01463848_m1, Rn01637087_m1, Rn01535512_g1, Rn01500392_m1 and Rn99999017_m1, Applied Biosystems) were detected in terms of commercially available TaqMan Expression Assays per manufacturer's instructions using Bio‐Rad CFX96 Touch Real‐Time PCR Detection System (Bio‐Rad, Hercules, California). For the assay, the TaqMan Universal PCR Master Mix (4 304 437, Applied Biosystems) was used. Samples were assayed in duplicates. Expression levels of mRNA were normalized to 18s rRNA (Applied Biosystems).

### 2.5 Histology

After sacrifice, whole spines were explanted and fixed in 10% neutral‐buffered formalin and decalcified in 0.5 M EDTA solution with weekly changes for 30 days. After decalcification, the spine was cut in the middle of the vertebral bodies from lumbar spine L4 until L6, resulting in single vertebral segments. Vertebral segments were bisected in the mid‐sagittal plane, processed for paraffin embedding and sectioned to a thickness of 5 μm. Sections were deparaffinized in a sequence of xylene, ethanol, and distilled water solutions. Then, the sections were stained with Hematoxylin and Eosin (H&E) or Alcian Blue/Picrosirius Red, containing Alcian Blue staining solution (pH 2.5) in 0.5% aqueous acetic acid (Sigma), and Picrosirius Red staining solution (0.1 g Sirius red/100 mL picric acid). Stained slides were scanned with an Aperio R slide scanner (Leica Microsystems) and analyzed for evidence of changes in the NP and AF using QuPath (software info). Morphological features evaluated included lesion of the NP, the sharpness of the boundary between the NP and the AF, and disruption of the AF.

### 2.6 Biobehavioral testing

The Randall‐Selitto and von Frey tests were was used as complimentary measures of mechanical sensitivity. The von Frey test is frequently used in similar studies that test pain behavior in rat models for IVD degeneration.^18^, ^40^, ^41^ While von Frey filaments detect tactile sensitivity, the Randall‐Selitto test allows to assess response thresholds to deep mechanical pressure.^44^ The Rotarod assay was included as a measure of motor capacity. Behavioral testing was conducted by a treatment‐blind experimenter between 3:00 pm and 7:00 pm in the Biobehavioral Research Core at Cedars‐Sinai. On each testing day, rats were brought into the Biobehavioral Research Core at least 30 minutes prior to the test session in order to habituate them to the environment. For Rotarod testing, movement‐evoked pain was assessed during ambulation on a Rotorod (San Diego Instruments). The rats were placed on the rotating rod for a 210‐seconds trial repeated three times at 30 minutes interval. For each trial, the rod was set at a start speed of 3 rpm that remained constant for 30 seconds, then, the rod gradually accelerated from 3 rpm to 30 rpm over a 3 minutes period. The latency to fall off the rod was averaged across the trials. For the Randall‐Selitto test, The Ugo Basile Analgesy‐Meter (www.ugobasile.com) was used to measure mechanical hyperalgesia,^45^ as described previously.^46^ The experimenter gently restrained the rat in one hand for testing on the paw pinch apparatus and with the other hand guided the hind paw to be tested on the plinth under the cone‐shaped pusher. A weight operated by the experimenter pressing a pedal‐switch exerted a force at a constant rate of 16 g per second. When the rat elicited paw withdrawal or showed nocifensive behavior, the experimenter released the pedal and recorded the applied force. Three measures for each paw were collected then averaged. For von Frey testing, an electronic von Frey (www.iitcinc.com) was used to assess mechanical/tactile allodynia as previously described.^47^ The animals were placed in a plexiglas testing chamber (22 cm × 22 cm) with a grid mesh floor. After a 15 minutes habituation period, a mechanical stimulus was delivered by applying a von Frey hair alternately under the plantar surface of the left and right hindpaws. The force necessary to produce paw withdrawal or nocifensive behavior was recorded. Von Frey testing was carried out immediately following the Randall‐Selitto test. For both the Randall‐Selitto test and von Frey testing the first paw to be assessed was randomly selected to avoid anticipation by the animal. Paw withdrawal thresholds were determined for left and right. For data evaluation, withdrawal thresholds from left and right were averaged and normalized to the preoperative baseline values of each animal, in order to highlight the effect of the IVD degeneration and to take into account inter‐animal variability.

### 2.7 Statistics

Prior to study start, the sample size was determined using G* Power software. The results indicated the following: to observe a 1‐fold difference at a p value of 0.05 with a power set to 80%, our needed sample size was 16. To account for any possible variation, we increased our sample size by 50%. The unit of observation for gene expression and MRI analysis was disc and for biobehavioral tests rat. Both L4‐5 and L5‐6 discs were kept in the same analysis. Variation due to disc level (L4‐L5 vs L5‐6) was evenly distributed across the testing groups to account for differences in the different spinal disc levels. All statistical analyses were performed using Prism 7 (GraphPad Software, Inc., La Jolla, California); *P* < .05 was considered to be statistically significant. The outcome measurements were (a) μMRI measures, (b) levels of gene expression, and (c) biobehavioral measures. To minimize inter‐animal variability, changes of the normalized results with time within and across experimental groups were analyzed. Separately for each dependent outcome measure, analysis of variance (two‐way analysis of variance [ANOVA]) was performed. Mean values were compared across experimental groups; for multiple comparisons, Tukey's post hoc test was used. Regression analysis of μMRI values was used to describe the relationship of IVD degeneration as a function of needle diameter (mm) that was used for injury. In figures, results are presented as mean values and bars indicate standard deviations (SD), except for biobehavioral tests, where the bars indicate SE of the mean.

## 3 RESULTS

### 3.1 Injury with a 18G needle results in the most severe disc dehydration

Micro‐MRI imaging analysis showed the most severe reduction in disc size (T1‐weighted scans) and hydration (T2‐weighted scans) in response to injury with a 18G needle‐the largest diameter needle shaft (Figure 2A).

To investigate changes in disc morphology and hydration of the nucleus pulposus in response to different needle injuries quantitatively, area values of T1‐weighted and of T2‐weighted scans of the IVDs of L4‐5 and L5‐6 combined were evaluated at 0, 4, and 8 weeks. When injured with a 18G needle, relative T1‐weighted area values of IVDs were decreased at both time points compared to week 0. Interestingly, the T1‐weighted area in the sham group was slightly increased at week 4 vs week 0, and at week 8 vs 0. Comparing between groups at the same time points, injury with a 18G needle resulted in lower T1‐weighted area values compared to sham control, to 21G and to 23G groups at 4‐ and 8‐ weeks postinjury. Furthermore, a decrease in relative T1‐weighted area values in the 21G and 23G group vs sham control was detected at week 4 only (Figure 2B, P values are shown in the figure).

To investigate changes in hydration of the nucleus pulposus in response to different needle injuries, area values of T2‐weighted scans of the IVDs of L4‐5 and L5‐6 combined were evaluated at weeks 0, 4, and 8. When injured with a 18G needle, relative T2‐weighted area values of IVDs were decreased at week 8 compared with week 0. When comparing between groups at the same time points, injury with a 18G needle resulted in lower T2‐weighted area values compared to sham control and 23G groups. Furthermore, a decrease in relative T2‐weighted area values in the 21G group vs sham control was detected at weeks 4 and 8 (Figure 2B, P values are shown in the figure).

Correlation analysis between T1‐ and T2‐weighted values of injured discs vs needle diameter (0.64 mm/23G, 0.82 mm/21G, and 1.27 mm/18G) was performed. Results demonstrated reduced T1‐weighted and reduced NP high intensity area values (T2‐weighted) with increasing needle diameters at weeks 4 and 8 postinjury (T1, week 4: *r* ^2^ = .47, *P* < .0001; T1, week 8: *r* ^2^ = .44, *P* < .0001; T2, week 4: *r* ^2^ = .19, *P* < .05, T2, week 8: *r* ^2^ = .17, *P* < .05, Figure 2C).

### 3.2 Gene expression of IVD degeneration markers is regulated in response to injury with 18G and 21G needles

**FIGURE 3:**
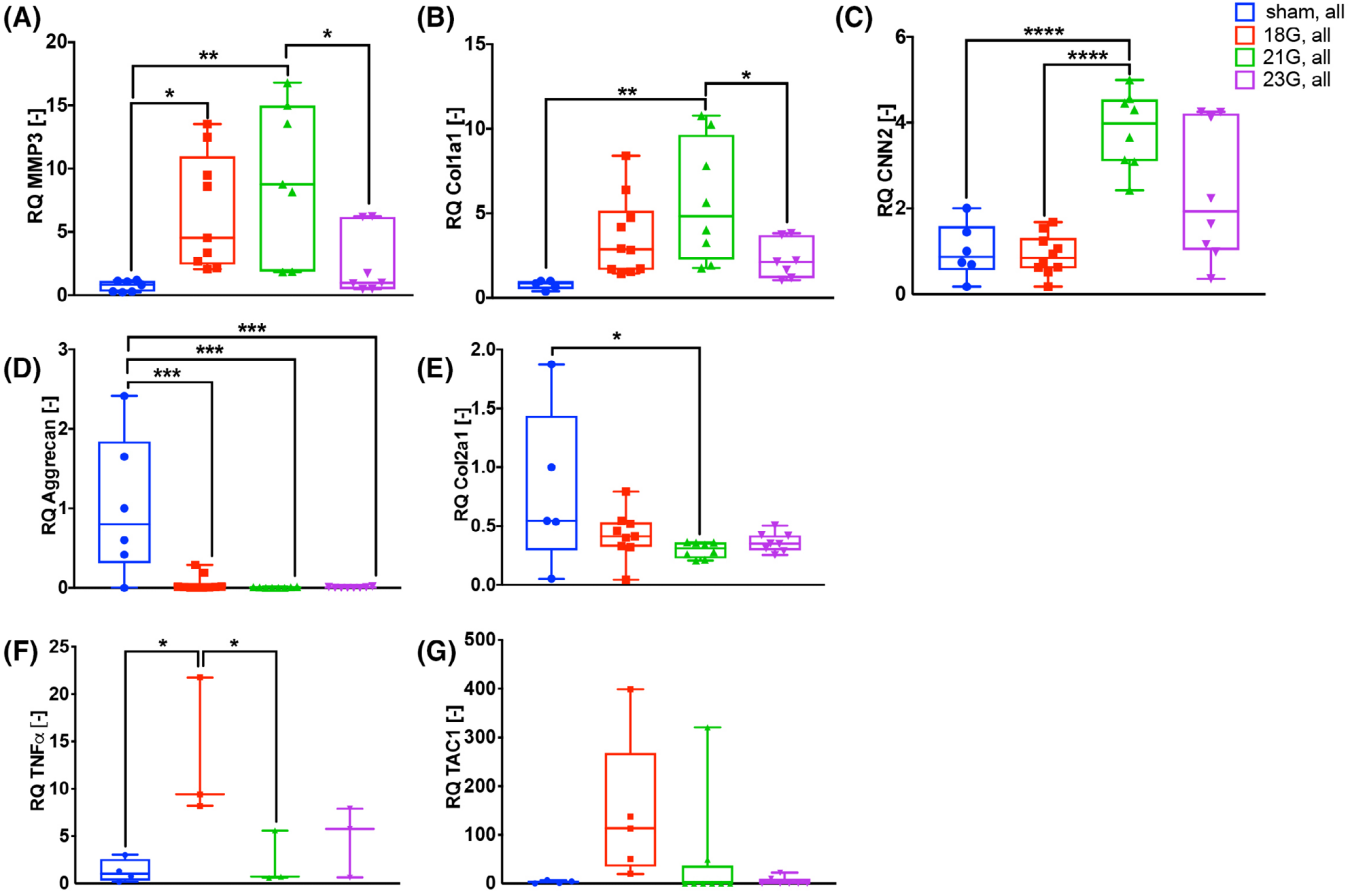
Gene expression of anabolic and catabolic markers are regulated in 18G‐ and 21G‐injured discs, while pain markers are increased in 18G‐injured IVDs only. Shown are the results from injured discs and matching uninjured discs from rats undergoing sham surgery. A‐C, MMP3 expression is upregulated in the 18G and 21G group, and collagen I and CNN2 are upregulated in 21G group. D,E, Aggrecan is downregulated in all injured groups, and collagen II in 21G. F,G, TNFα is upregulated in the 18G group. Results are displayed for L4‐5 and L5‐6 combined IVDs (sham, 18G, 21G, and 23G injured groups). *P < .05, **P < .01, ***P < .001; ****P < .0001

To investigate changes in gene regulation in IVDs in response to different needle injuries, quantitative RT‐PCR of the IVDs of L4‐5 and L5‐6 combined were evaluated after the animals' sacrifice at week 8. An upregulation of the catabolic marker, MMP3, was detected in the 18G and 21G groups vs sham control. Furthermore, MMP3 levels were elevated in the 21G vs the 23G group (Figure 3A). Collagen I levels, an indicator of fibrotic activity in the disc were increased in the 21G group vs sham group as well as vs 23G (Figure 3B). Gene expression of CNN2, another marker for fibrotic activity, was upregulated in 21G group compared to sham control and 18G (Figure 3C). Expression of the main NP matrix gene, aggrecan, was downregulated in all injury groups compared with sham control (Figure 3D). Expression of collagen II was downregulated in the 21G group vs sham control only (Figure 3E). The neuropeptide gene, TAC1, was not significantly regulated (Figure 3F). TNF‐α was only detectable in a subset of the samples analyzed. However, an upregulation in the 18G group compared to sham control and 21G groups was found (Figure 3G).

### 3.3 Histo‐morphological analysis shows most severe NP degeneration in response to 18G injury

**FIGURE 4:**
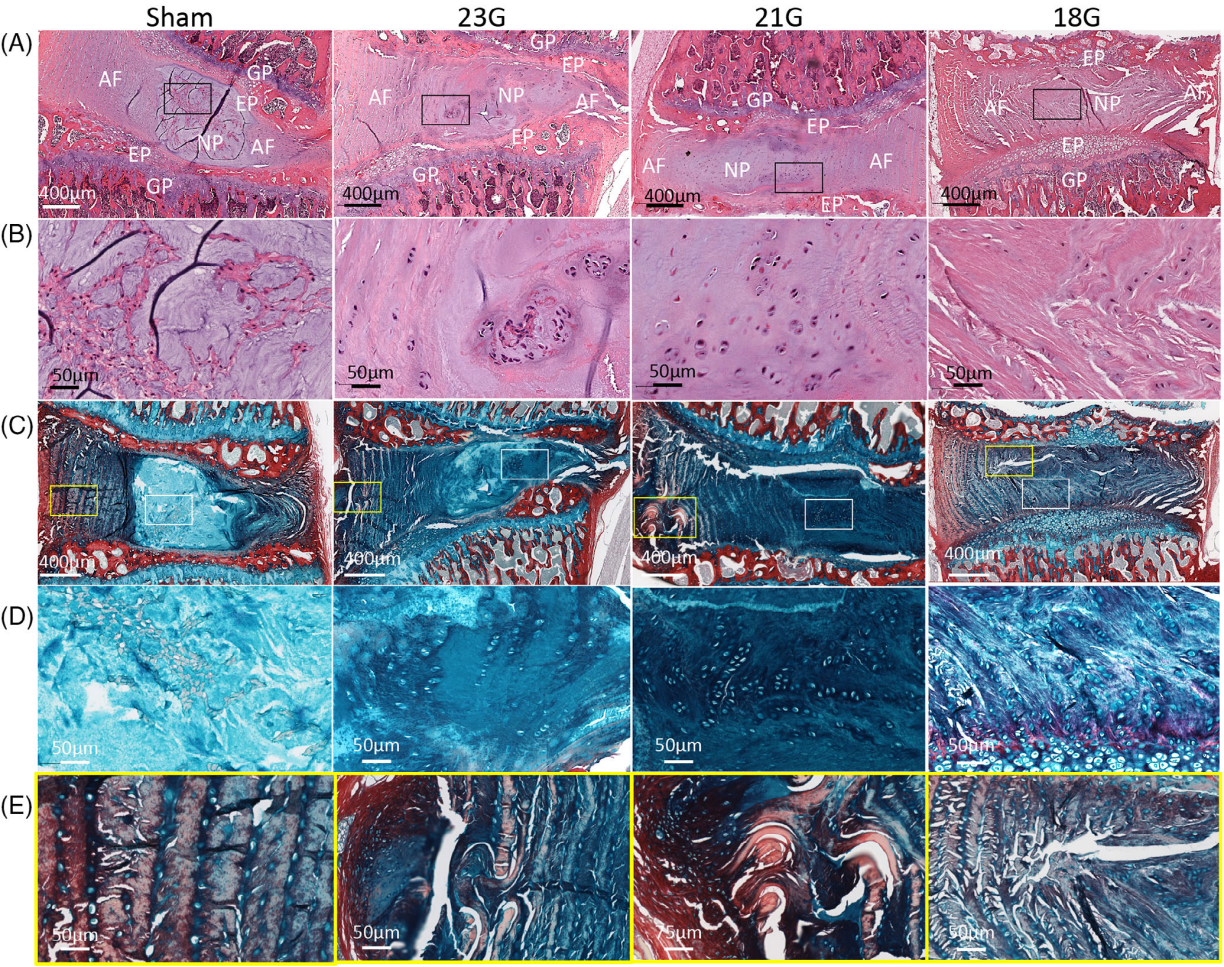
IVD degeneration and NP/AF lesion in response to needle puncture injury is most pronounced in the 18G group. A, H&E stained lumbar discs from sham and 18G, 21G, and 23G injured groups, explanted at 2 months postsurgery. B, Magnification of the NP site represented by black rectangle on row. C, Alcian blue/Picrosirius Red stained lumbar discs from sham and 18G, 21G, and 23G injured groups, explanted at 2 months postsurgery. D, Magnification of the NP site represented by white rectangle on row. E, Magnification of the AF needle insertion site represented by yellow rectangle on row. NP, nucleus pulposus; AF, annulus fibrosus, EP, end plate, GP, growth plate

H&E and Alcian Blue/Picrosirius Red staining showed a complete disappearance of the NP and the NP‐AF interface in the 18G needle group, and a partial deformity of the NP in the 21G group (Figure 4A‐E). In the 23G group, the NP‐AF interphase was relatively intact. In all injury groups, the morphology of NP cells showed major changes compared with sham controls. The clusters of small cells surrounded by cell‐free matrix, characteristic for healthy NP, are not visible in any of the injured groups. Instead, islands of remaining NP tissue are evident in the 23G group and rows or clusters of large proliferating cells in 21G and 18G groups. In the 18G group, very little of remaining NP tissue can be observed. It is mainly replaced by fibrotic tissue, similar to the disrupted AF tissue and low in GAGs, as visible by a lighter Alcian Blue staining (Figure 4C,D). At the needle insertion site, the AF was disrupted in all injury groups (Figure 4E).

### 3.4 Mechanical sensitivity is increased in response to IVD injury with a 18G needle

**FIGURE 5:**
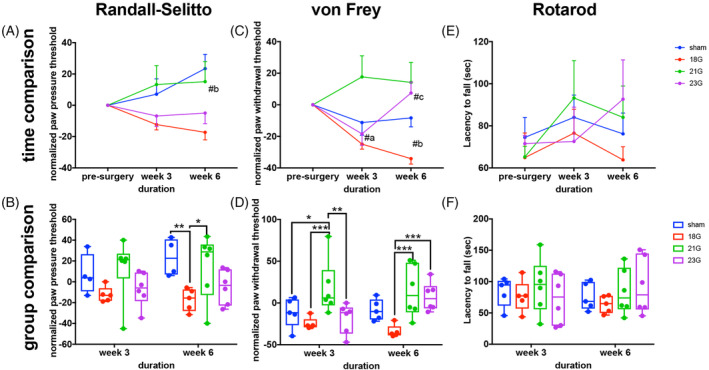
Biobehavioral tests show increased sensitivity to paw pressure (Randall‐Selitto) and mechanical stimuli (von Frey) in rats injured with a 18G needle. The results of, A,B, Randall‐Selitto, C,D, von Frey and, E,F, Rotarod biobehavioral tests of rats injured with 18G, 21G, and 23G needles, performed presurgery as well as at weeks 3 and 6 postsurgery are shown. Top, A,C,E, differences between time‐points are shown. Bottom, B,D,F, differences between experimental groups are shown. *Indicates significant differences between experimental groups: #a: pre vs 3 weeks, #b: pre vs 6 weeks, #c: 3 vs 6 weeks *P < .05, **P < .01, ***P < .001

Longitudinal analysis with the Randall‐Selitto device demonstrated an increased normalized paw withdrawal threshold in the sham group at week 6 vs presurgery (Figure 5A). Comparison between groups 6 weeks after injury showed a reduced normalized withdrawal threshold in the 18G needle group compared with the sham and 21G groups (Figure 5B). Regarding the longitudinal von Frey testing, a reduction in the normalized paw withdrawal threshold of 18G needle injured animals was detected at weeks 3 and 6 post‐injury compared to pre‐surgery. Furthermore, an increase was detected in the 23G group at week 6 vs week 3 (Figure 5C). In the group comparison, at week 6, the normalized paw withdrawal threshold of the 18G needle group was lower than 21G and 23G group (Figure 5D). Rotarod evaluation detected no differences in the rats' latency to fall over time or between groups (Figure 5E,F).

## 4 DISCUSSION

This is the first study using multiple assessment methods to demonstrate the responses in rat lumbar IVD degeneration, pro‐nociceptive marker expression, and pain‐related behavior to IVD injury with increasing needle diameters (decreasing gauge). Our results show a correlation between the needle diameter and the hydration of lumbar IVD. Injury with a 18G needle, the largest diameter, had the strongest IVD degeneration‐inducing effect, as demonstrated by the greatest loss of NP water content and most severe lesion of the AF and NP, it had the biggest impact on pro‐nociceptive mRNA marker regulation, and it increased sensitivity to mechanical stimuli. Catabolic, anabolic and fibrosis gene marker regulation in the IVD was detected in both the 18G and 21G needle injury group.

Micro‐MRI analysis demonstrated a reduction in disc area (T1‐weighted) and NP hydration (T2‐weighted) values in the 18G needle group compared to sham control and to 23G needle groups at weeks 4 and 8 postsurgery. Furthermore, a correlation between needle diameter (mm) and disc area as well as NP hydration values was detected. Our results are in line with studies in rat caudal spines showing an induction of disc degeneration in response to disc puncture with 18G and 21G needles.^35^, ^36^, ^48^ For example, a reduced X‐ray‐based disc high index and poorer disc hydration in rat caudal spines injured with an 18G compared to 25G needle and uninjured controls was shown.^36^ Quian et al. demonstrated an induction of IVD degeneration at 2 weeks postinjury in a rat tail model injured with a 18G, but not a 26G needle, using T2‐weighted imaging.^49^ Furthermore, a significant reduction in T2 density and MRI index in response to rat caudal spine injury with a 18G or 21G needle compared to intact controls was shown.^35^

To our knowledge there is no prior study comparing the effect of different needle diameters on the induction of IVD degeneration in rat lumbar discs, although the degree of lumbar disc degeneration is strongly associated with degenerative disc disease, a common cause of low back pain.^50^ To induce IVD degeneration in rat lumbar spine, needles of varying diameters have been used.^18^, ^40^, ^41^, ^42^ For example, a recent study by our group demonstrated the successful induction of IVD degeneration in lumbar rat spine by inserting a 18G needle in a retroperitoneal approach, which resulted in a similar reduction of IVD hydration using μMRI compared to the present study.^42^ Also in line with our study, rat lumbar disc injury with a 21G needle resulted in an increase in IVD degeneration compared to pre‐surgical controls, as demonstrated via MRI and H&E staining.^40^ In contrast, a study by Li et al showed injury of lumber discs with a 27G needle to be sufficient to create a slight lesion of the NP and to induce loss of disc hydration at week 8 postsurgery.^38^ While the histological findings are in line with our findings, our study results do not confirm that a single puncture with a needle ≥23G is sufficient to induce loss of IVD hydration in the same model system. When using a small needle diameter, a more efficient induction of IVD degeneration might be achieved in a combinatory approach with intradiscal PBS or TNF‐α injections, as demonstrated in prior research.^18^, ^41^

It is noteworthy that no clear signs of IVD degeneration were detected in the 23G group. For cell therapeutic injections into the IVD, use of very small needles can create high shear stress and consequent low cell viability or high viscosity.^51^ Therefore, the maximal needle size for therapeutic injections is important to know. Based on our data we suggest choosing a maximal needle size of 23G for this purpose.

Our study showed an upregulation of MMP3 mRNA expression in response to both injuries with a 18G and 21G needle, but not 23G. Similar to our findings, MMP‐3 has been shown to be increased in severely but not mildly degenerated human NPs.^52^ Furthermore, a study by Yang et al. detected a decrease in aggrecan gene expression levels in mouse injured tail discs at 2, 6, and 12 weeks postinjury.^53^ In contrast, no downregulation of aggrecan gene expression was detected in response to rat caudal tail injury, using a 18G needle.^36^ Our study detected a decrease in collagen II and increase in collagen I and CNN2 gene expression levels in the 21G group only. In human and animal degenerated NP tissues, a reduction in collagen II and abnormal deposition of collagen 1 have been shown.^11^, ^54^ Similarly, in rat injured caudal IVDs, an upregulation of collagen I and downregulation of collagen 2 has been detected at 2, 4, and 8 weeks postinjury.^36^, ^55^ Furthermore, increased CNN2 expression in degenerated human IVDs has been detected.^56^ We observed that fibrosis markers expression (collagen I and CCN2) was clearly enhanced in the 21G vs 18G group. The reduced collagen I and CCN2 gene expression levels in the 18G group may be a result of enhanced or accelerated cell apoptosis of NPCs due to the increased lesion of the NP.^55^ However, this should be investigated in more detail in the future.

Assessment of TNFα gene expression levels in injured vs noninjured discs showed increased expression levels in the 18G group vs control. Our findings on TNFα expression are consistent with a study of Evashwick‐Rogler et al showing increased TNFα levels in injured rat IVDs.^18^ Moreover, TNFα‐immunoreactivity positively correlated with IVD degeneration and negatively with normalized paw withdrawal thresholds.^18^ In this study, TNFα gene overexpression was only observed in a subset of samples following disc injury. While increased TNFα levels have been frequently shown to be associated with the development and progression of IVD degeneration, and with discogenic pain in rat models, evidence is usually provided in the literature on a protein level.^18^, ^55^, ^57^ The observed differences might be therefore due to differences in TNFα gene and protein expression at the time point investigated.

Pain behavior assessments using von Frey showed behavioral signs of mechanical hypersensitivity at 6 weeks postsurgery vs presurgery in rats that were injured with an 18G needle, but not with a 21G needle in our study. The increases in pain sensitivity could either be due to pain primarily from the painful degenerating disc (discogenic pain) but could also be from increased radicular (nerve) pain from compression due to increased spinal central canal and lateral recess stenosis secondary to intervertebral disc collapse.^58^ The dependence of pain behavior on the severity of IVD degeneration has been shown in a previous study that detected a more rapid development of behavioral changes (tested via pressure hyperalgesia directly over the punctured discs) in response to IVD injury of L4‐5 and L5‐6 in rat using a larger drill (0.8 mm, equivalent to 21G) vs a smaller drill (0.5 mm, equivalent to 25G).^59^ In line with our study, no reduction in von Frey 50% force withdrawal were detected in this study using those sizes.^59^ Furthermore, the lack of significant changes in the 21G group is in line with a study from Liu et al showing no changes in 50% withdrawal thresholds using von Frey in response to anterior lumbar disc puncture with a 21G needle compared with presurgery.^40^ In contrast, rat lumbar IVD injury using a 26G needle in combination with PBS and TNF‐α injections into the discs demonstrated a significant reduction of paw withdrawal thresholds normalized to presurgery using von Frey.^18^, ^41^ Randall‐Selitto behavior tests that were performed in our study showed a similar pattern as von Frey testing. Lack of significant differences between the sham and 18G group at 3 and 6 weeks using von Frey and between pre‐ and postsurgery in the 18G group using Randall‐Selitto testing may be a result of a limited sample number and small effect size and should be therefore tested with larger rat cohorts in the future. Furthermore, differences between von Frey and Randall‐Selitto may be a result of the mode of stimulation (tactile perception vs mechanical pressure). Different modes of stimulation may activate different nociceptors and/or mechanoreceptors, leading to different responses.^44^ The lack of differences in the animals' motor function in our model via Rotarod is in line with the literature showing no differences in motor function in a rat lumbar IVD injury model using a 26G needle in combination with PBS.^41^

In this model of IVD degeneration we utilize the rat lumbar intervertebral disc injury, which requires an open anterior surgical approach and is more invasive compared to the rat‐tail model. We believe that the lumbar model more accurately represents the human lumbar IVD condition, as rat tails do not have facet joints and have significantly different mechanical and viscoelastic properties.^60^ Moreover, the highly gelatinous NP of rat caudal IVDs may limit the translation of these findings to human IVDs, which contain a more fibrous NP tissue.^61^ Beckstein et al demonstrated that the disc tissue material properties are similar between rat and human disc after adjusting for geometry, despite differences in GAG and water composition.^62^

This study is not without limitations. Firstly, time points for imaging and biobehavioral testing did not match, which makes it difficult to compare these outcome measures. Secondly, the study's end point chosen was 8 weeks. The early time‐points of 3 to 4 weeks selected in this study have relevance for an acute model, while later time points of 4 to 5 weeks rather simulate intermediate (to chronic) and already painful disc degeneration.^59^, ^63^ Based on a study by Leimer et al, studying long term effects and chronic back pain development may require longer follow‐up of 16 to 20 weeks. The changes in our injury model were evident within the frame of the study and testing of future therapeutics could be applied in the acute or intermediate phase. However, we are not providing a model for chronic disc degeneration and our animal model does neither mimic the onset nor the chronic stage of disc degeneration in humans in its full complexity. Thirdly, our biobehavioral tests were limited. There are additional pain modalities like cold sensitivity that have been published before in various mouse models.^64^, ^65^, ^66^ Furthermore, more direct assays of axial low back pain, such as tail suspension testing as well as nonstimulus evoked tests, such as gait analysis in an open field test should be considered in the future for behavioral data validation.^44^ Future studies should also look at the effect of the IVD injury on sensory neurons residing in the DRG and the spinal cord plasticity as a result of the injury and/or intervention. Finally, nerve or vascular in‐growth into the IVD should be studied. There is no doubt that nervous system plays an active role in backpain generation. Therefore, deeper understanding of the underlying mechanisms that mediate the interaction between the sensory neurons and IVD cells is needed.

In conclusion, we show a method to reliably modulate the degree of lumbar IVD degeneration and associated pain responses by varying the size of the needle puncture. This will allow us to mimic the spectrum of IVD degeneration seen in the human population, which can range from mild to severe. In our study, IVD puncture with a 21G needle clearly demonstrated signs of moderate disc degeneration, but we did not show reliable pain responses when using this needle. This model may be utilized for testing of cell‐based therapies and other therapeutic agents in order to prevent discogenic pain, since the ORS Spine Section emphasized the need of selecting moderate severity disc degeneration and avoiding too mild or too advanced degeneration.^23^ Injury with a 18G needle induced severe disc degeneration, associated with discogenic pain in our study. Our model may be used to study therapeutic agents for treatment of discogenic pain, which may be applied in combination with stem cell therapies and biomaterials approaches, to repopulate the disc space with viable cells and to restore the structural integrity of the disc. Prior to this several things should be considered: To elucidate the effect of needle injury on IVD function and pain behavior in more depth, more diverse biobehavioral assays (eg, open field, tail suspension or grip force tests) as well as biomechanics assessment of the IVD segments (eg, motion testing) should be included. In addition, detection of quantitative changes in IVD glycosaminoglycans (GAGs) in response to needle injury should be considered, since GAGs are known to be reduced during early disc degeneration.^67^ Finally, a combinatory approach of a 21G needle with a pro‐inflammatory agent, such as TNF, should be considered to explore the options to achieve a uniform moderate degeneration and consistent behavioral pain responses.

## CONFLICT OF INTEREST

The authors have no conflict of interest.

## AUTHORS CONTRIBUTION

Juliane D Glaeser, Wafa Tawackoli, Laura S. Stone, Hyun W. Bae, and Dmitriy Sheyn contributed to the concept of the study. Juliane D Glaeser, Wafa Tawackoli, and Dmitriy Sheyn designed the experiments. Juliane D Glaeser, Wafa Tawackoli, Derek G Ju, Khosrowdad Salehi, and Zachary NaPier performed animal procedures. Juliane D. Glaeser and Jae H. Yang performed the MRI analysis. Khosrowdad Salehi, Victoria Yu, Evan Saidara, and Zhanna Khnkoyan performed all the RT‐PCR and immunostaining. Jean‐Phillipe Vit performed the biobehavioral studies. Juliane D. Glaeser, Linda EA Kanim, and Dmitriy Sheyn performed the statistical analyses. Juliane D. Glaeser, Derek G. Ju, Laura S. Stone, and Dmitriy Sheyn wrote the manuscript.
